# Prognostic Nutritional Index identifies risk of early progression and survival outcomes in Advanced Non-small Cell Lung Cancer patients treated with PD-1 inhibitors

**DOI:** 10.7150/jca.55936

**Published:** 2021-03-15

**Authors:** Na Liu, Aimin Jiang, Xiaoqiang Zheng, Xiao Fu, Haoran Zheng, Huan Gao, Jingjing Wang, Xuan Liang, Tao Tian, Zhiping Ruan, Yu Yao

**Affiliations:** Department of Medical Oncology, The First Affiliated Hospital of Xi'an Jiaotong University, No.277 Yanta West Road, Xi'an, Shaanxi 710061, People's Republic of China.

**Keywords:** non-small cell lung cancer, prognosis nutritional index, PD-1 inhibitors, prognosis, nomogram

## Abstract

**Background:** The prognostic nutritional index (PNI) is related to the prognosis of multiple malignancies. This study investigated whether the PNI has prognostic value in advanced non-small cell lung cancer (NSCLC) patients treated with programmed death 1 (PD-1) inhibitors.

**Methods:** We retrospectively analyzed advanced NSCLC patients treated with PD-1 inhibitors from July 2018 to December 2019. Pretreatment PNI was calculated by peripheral lymphocyte count and serum albumin level, and the cut-off value was determined. Subsequently, we investigated the relationship between PNI and early progression, and evaluated its prognostic role on survival outcomes. Ultimately, based on the results of survival analysis, a nomogram was established.

**Results:** A total of 123 patients were included. Of these, 24 (19.5%) patients had experienced early progression. Multivariate logistic analysis indicated that low PNI (odds ratio, 3.709, 95% confidence interval [CI], 1.354-10.161; *P* = 0.011) was closely correlated with early progression. Moreover, multivariate Cox regression analysis confirmed that low PNI was an independent risk factor for progression-free survival (hazard ratio [HR], 2.698, 95% CI, 1.752-4.153; *P* < 0.001) and overall survival (HR, 7.222, 95% CI, 4.081-12.781; *P* < 0.001), respectively. The prediction accuracy of nomogram based on PNI is moderate.

**Conclusion:** PNI was an independent predictor of early progression and survival outcomes in advanced NSCLC patients treated with PD-1 inhibitors.

## Introduction

According to cancer statistics, 2020, lung cancer (LC) is the second incidence rate and the first death rate cancer among men and women, accounting for 13% of all cancer diagnoses and 23% of all cancer-related deaths 1. Non-small cell lung cancer (NSCLC) accounts for more than 85% of LC, and the 5-year overall survival (OS) rate in advanced stage patients is less than 5% 2. In recent years, with the improvement of biological understanding of LC and the remarkable progress of immunotherapy in LC, the OS of advanced NSCLC patients has been prominently improved.

Immune checkpoint inhibitors (ICIs) have been applied in the clinical practice. Early-stage clinical trials have shown that approximately 14%-20% of advanced NSCLC patients receiving ICIs had a rapid and sustained response. The results of KEYNOTE-024 and KEYNOTE-042 trials suggested that pembrolizumab monotherapy was approved for the first-line treatment in patients with programmed death ligand-1 (PD-L1) expression ≥ 50% 3, 4. However, according to the results of KEYNOTE-189 and KEYNOTE-407, patients can benefit from pembrolizumab plus chemotherapy regardless of their PD-L1 expression status 5, 6, 7. In the CheckMate-227 trial, patients with high tumor mutation burden (TMB) could benefit from nivolumab combined with ipilimumab, and TMB may be an ideal predictor in these individuals. However, this study also showed that the clinical benefit of patients was not related to PD-L1 expression status. Analysis of blood TMB (bTMB) in POPLAR and OAK trials indicated that patients with high bTMB could benefit more from atezolizumab 8, 9. Due to the lack of correlation between TMB and PD-L1 expression 10, 11, the combination may not exert a synergistic effect. Currently, immunotherapy still lacks effective biomarkers. PD-L1, TMB, tumor-infiltrating lymphocytes (TILs), and mismatch repair (MMR) cannot be used as golden indicators for choosing the benefit population.

Recently, mounting studies have highlighted the significance of nutritional and immune status in oncological patients, suggesting that it is pivotal in cancer progression and prognosis 12, 13. Prognostic nutritional index (PNI) is obtained by the level of serum albumin and peripheral lymphocytes, which can reflect the nutritional and immune status of patients 14. Several studies have confirmed that PNI is related to the response and prognosis of patients with chemotherapy or chemoradiotherapy 15, 16. Moreover, the indicator is readily available, non-invasion and dynamically monitored, with a high clinical application prospect. However, whether PNI is related to the clinical outcomes of advanced NSCLC patients treated with programmed death-1 (PD-1) inhibitors remains unclear. Therefore, our study evaluated the impact of PNI on early progression and prognosis in those patients.

## Methods

### Patient selection

We collected the clinical information of advanced NSCLC patients who received PD-1 inhibitors in the First Affiliated Hospital of Xi'an Jiaotong University from July 2018 to December 2019. The inclusion criteria were as follows: (1) age over 18 years old; (2) patients who were diagnosed by histopathology or cytopathology as NSCLC; (3) stage IIIB-IV treated with PD-1 inhibitors; (4) Eastern Cooperative Oncology Group Performance Status (ECOG PS) score at 0-2; (5) all patients had available hematological parameters (peripheral lymphocyte count and serum albumin level) and evaluable imaging data before treatment; (6) at least one cycle of PD-1 inhibitors treatment (monotherapy or combination therapy). The exclusion criteria were: (1) patients with incomplete clinicopathological data and follow-up information; (2) combined with autoimmune diseases, hematologic disorders, and other diseases; (3) a history of using steroid within two weeks; (4) infectious diseases before PD-1 inhibitors treatment; (5) patients with other primary carcinomas. The study was approved by the Ethics Committee of our hospital (XJTU1AF2020LSK-141).

Basic clinicopathological data were collected from patients who correspond to the standard. Clinical responses were assessed according to iRECIST criteria. Early progression was defined as progression or cancer-related death occurring within 8 weeks after receiving PD-1 inhibitors. Progression-free survival (PFS) was calculated from the start of treatment with PD-1 inhibitors to disease progression or death. And OS was calculated from the start to death for any cause. The last follow-up was performed on August 30, 2020.

### Prognostic nutritional index

The calculation formula of PNI was: serum albumin (g/L) + 5 × peripheral lymphocyte count (10^9^/L), and the results within one week before treatment were required. The receiver operating characteristic (ROC) curve was established, and the state variable value corresponding to the maximum value of Youden index was the PNI cut-off value. According to the cut-off value, patients were divided into L- and H- PNI groups.

### Statistical analysis

The Chi-square or Fisher's exact test was used to compare clinicopathological data between H-PNI and L-PNI groups. Continuous variables are compared using student's t-test. The relationship between clinical variables and the risk of early progression was assessed by logistic regression analysis. The survival curves of H-PNI and L-PNI groups were drawn by Kaplan-Meier (K-M) method. The Cox regression analysis was utilized to determine the prognostic factors of PFS and OS. Finally, a nomogram related to prognosis was developed, and the predictive accuracy of the model was assessed. All statistical analyses were two-sided probability tests (α = 0.05), *P* < 0.05 was considered the difference to be statistically significant. The above statistical analysis was performed using IBM SPSS Statistic 18.0 and R Studio 3.6.0.

## Results

### Patient characteristics

A total of 123 patients were enrolled in this study (Table 1). The average age at the time of diagnosis was 59.9 ± 11.3 years. Male patients account for the majority (79.7%). 60.2% of patients were lung adenocarcinoma (LUAD), 37.4% were lung squamous cell carcinoma (LUSC), and 2.4% were other types (2 cases of adenosquamous carcinoma, 1 case of large cell carcinoma). Stage IIIB/IIIC accounted for 18.7%. All of them had used at least one cycle of PD-1 inhibitors during the study period (17.9% for nivolumab, 42.3% for pembrolizumab, 25.2% for sintilimab, 8.1% for camrelizumab, and 6.5% for toripalimab), of which 42.3% were first-line therapy and 78% were combined chemotherapy. 27.6% of patients had known the PD-L1 expression status (1-49%, 12 cases; ≥ 50%, 12 cases; and negative, 10 cases). Patients with driver genomic alterations account for 17.1% (epidermal growth factor receptor [EGFR], 16.3%; ROS1 proto-oncogene receptor tyrosine kinase [ROS1], 0.8%). 13.0% of patients had liver metastases, 16.3% had central nervous system (CNS) metastases, and 30.9% had bone metastases. During the treatment, 24.4% of patients developed immune-related adverse events (irAEs).

ROC curve showed that the optimal PNI cut-off value was 46.05 (Supplementary Figure S1). The corresponding sensitivity and specificity were 0.833 and 0.683, respectively (area under the ROC curve was 0.780). According to the cut-off value (46.05), patients were divided into L-PNI (53 cases) and H-PNI (70 cases) groups. As shown in Table 1, the relationship between PNI and clinical characteristics of patients was summarized. The two groups had significant differences in CNS metastasis (*P* = 0.031) and early progression (*P* = 0.009).

### Early progression

**Figure 1:**
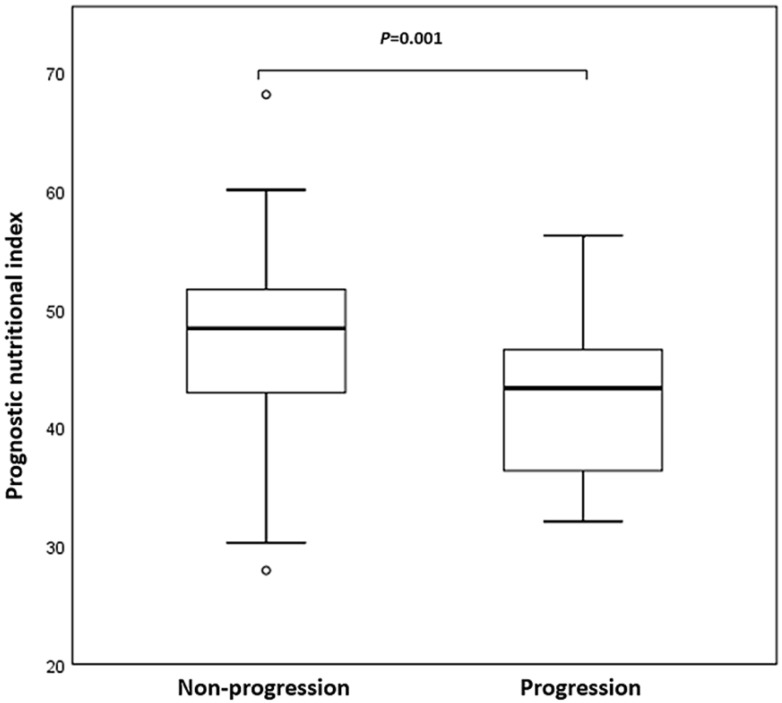
Box plot of prognostic nutritional index (PNI) in non-small cell lung cancer (NSCLC) patients with non-progression and early progression. Early progression was defined as progression within 8 weeks after initiation of programmed death 1 (PD-1) inhibitors.

Twenty-four patients (19.5%) had developed early progression (within 8 weeks). Figure 1 was a box plot of the relationship between early progression and PNI. Compared with non-early progression patients, the PNI level of patients with early progression was remarkably decreased (*P* = 0.001). Furthermore, logistic regression analysis showed that the therapy line, driver genomic alterations, and PNI were associated with early progression. The multivariate analysis confirmed that the therapy line (odds ratio [OR], 5.860, 95% confidence interval [CI], 1.544-22.246; *P* = 0.009) and PNI (OR, 3.709, 95% CI, 1.354-10.161; *P* = 0.011) were independent predictors of early progression.

### Survival analyses

**Figure 2:**
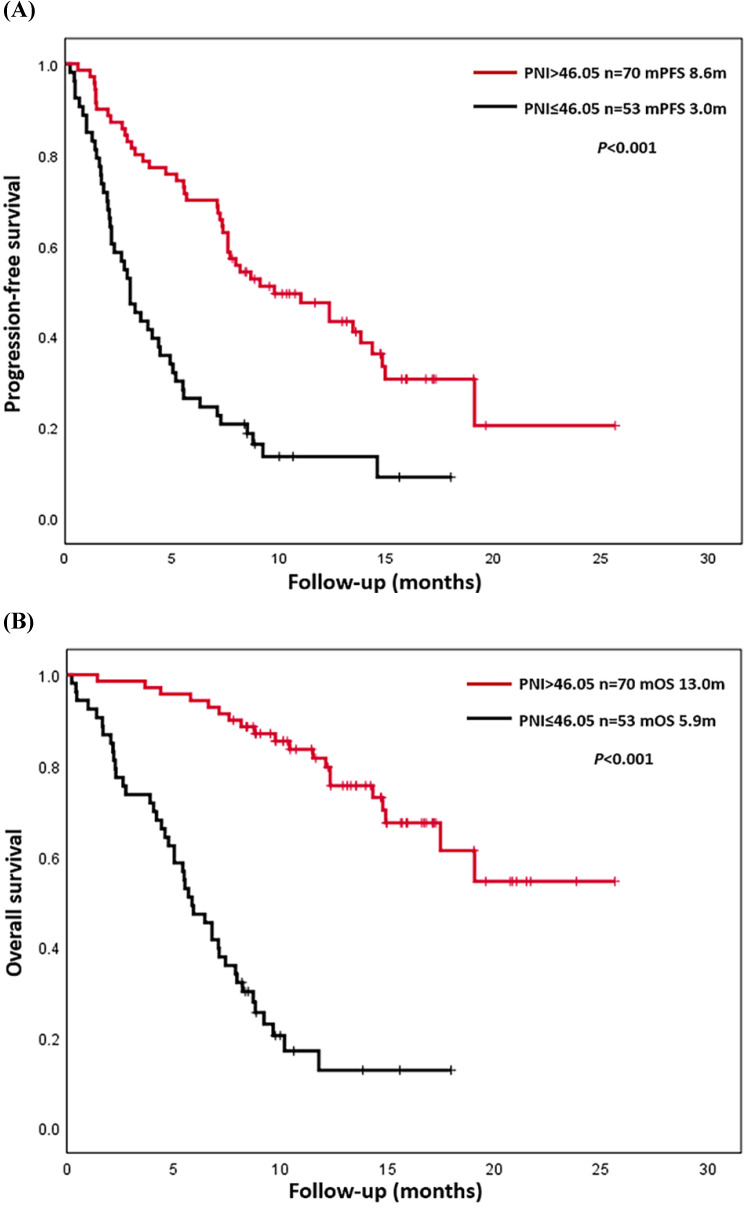
Kaplan-Meier analysis of progression-free survival (PFS) and overall survival (OS). (A) Final descriptive Kaplan-Meier estimates of PFS in all patients as well as stratified by high (>46.05) and low (≤46.05) prognostic nutritional index (PNI). (B) Final descriptive Kaplan-Meier estimates of OS in all patients as well as stratified by high (>46.05) and low (≤46.05) PNI.

Up to August 30, 2020, a total of 90 patients (73.2%) had progressed, and 63 patients (51.2%) had died. The causes of death were all related to lung cancer or its complications. The overall PFS was 0.2-25.7 months, and the median PFS (mPFS) was 7.1 months. The overall OS was 0.2-25.7 months, and median OS (mOS) was 12.3 months. K-M survival curves of PFS and OS were drawn according to L-PNI and H-PNI. The results suggested that PFS and OS in H-PNI patients were significantly higher than those in L-PNI patients (mPFS: 8.6 m vs. 3.0 m, *P* < 0.001; mOS: 13.0 m vs. 5.9 m, *P* < 0.001) (Figure 2). Also, we conducted K-M survival analyses of PFS and OS for LUAD and LUSC patients respectively (Figure S2), and the conclusion was consistent with the overall results.

The univariate analysis suggested that therapy line ≥ 2, accompanied by driver genomic alterations, bone metastasis, and L-PNI were associated with inferior PFS (Table 2). The multivariate analysis indicated that only PNI (hazard ratio [HR], 2.698, 95% CI, 1.752-4.153; *P* < 0.001) was an independent prognostic factor for PFS (Table 2). Similarly, ECOG PS score 1-2, therapy line ≥ 2, and L-PNI were related to poorer OS (Table 3). Further multivariate analysis showed that the therapy line (HR, 1.898, 95% CI, 1.108-3.254; *P* = 0.011) and PNI (HR, 7.596, 95% CI, 4.278-13.486; *P* < 0.001) were independent prognostic factors for OS (Table 3).

### Nomogram for overall survival

**Figure 3:**
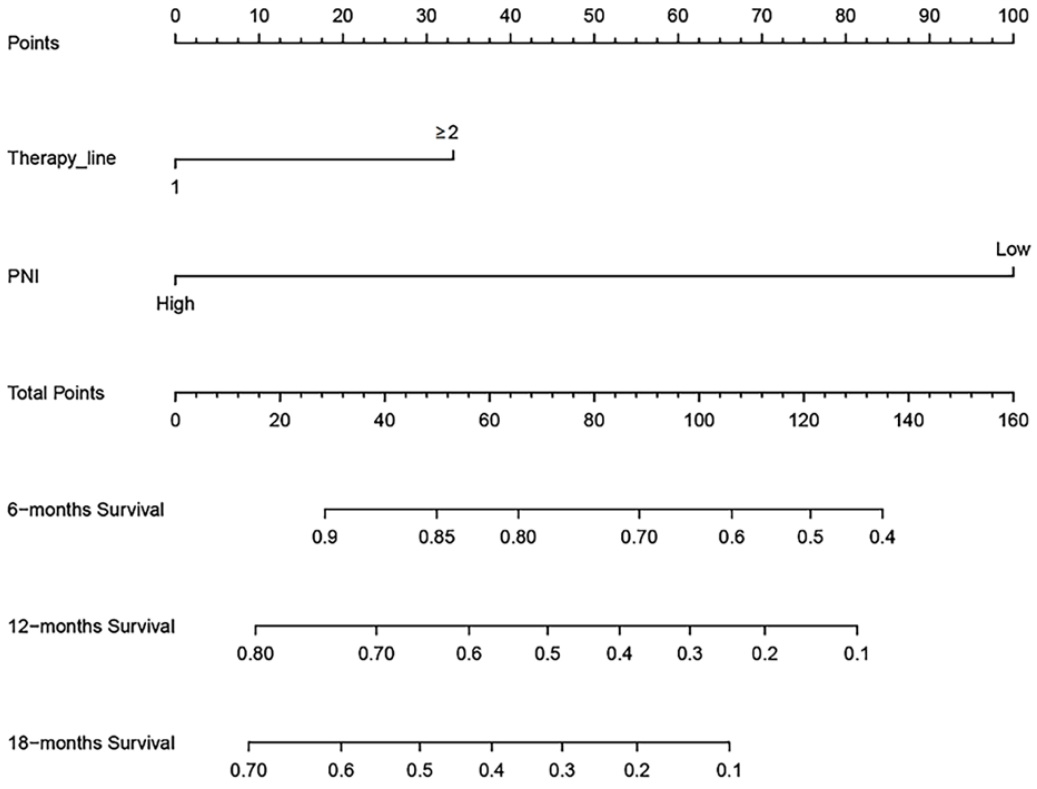
A survival nomogram for 6-, 12- and 18-months overall survival (OS) for non-small cell lung cancer (NSCLC) patients. Nomogram can be interpreted by adding up the points assigned to each variable. The total point projected on the bottom scale represents the probability of 6-, 12- or 18-months OS.

**Figure 4:**
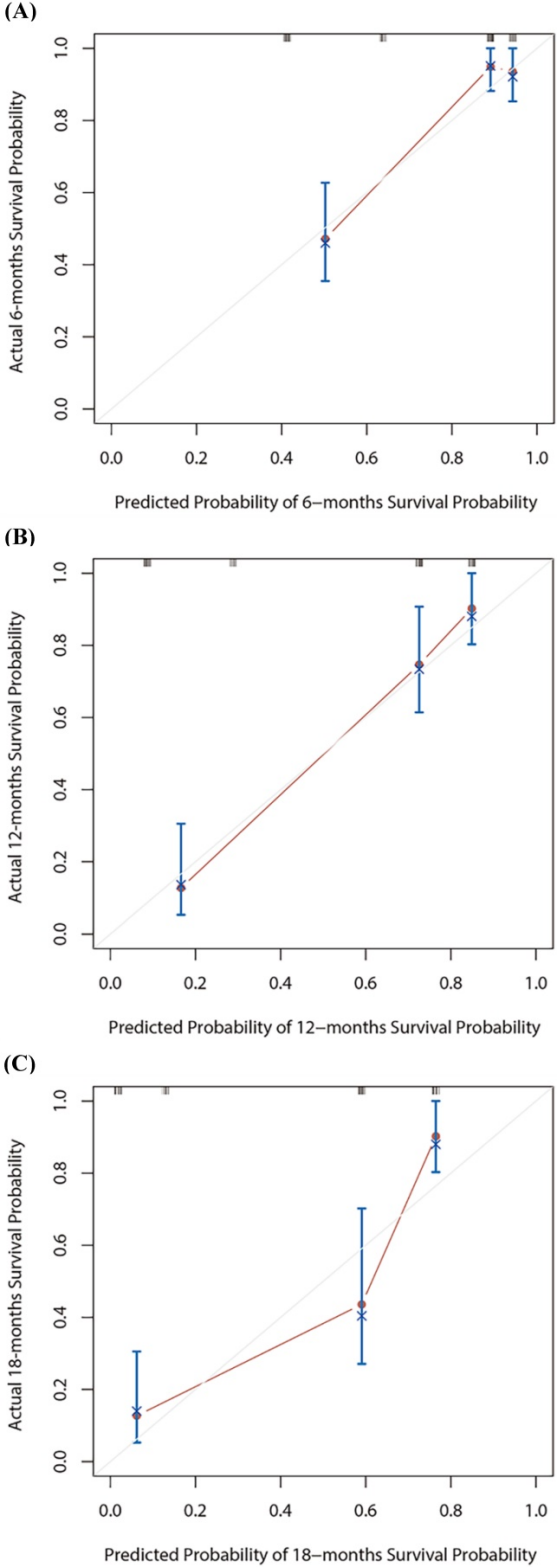
The calibration curves for 6-, 12- or 18-months overall survival (OS) nomogram (A-C). The X axis is nomogram predicted survival probability and Y axis is actual survival probability.

Based on the multivariate Cox regression analysis, we identified prognostic predictors (therapy line and PNI) for OS. And we selected them to construct a survival nomogram to predict the 6-, 12- and 18-months OS for advanced NSCLC patients who received PD-1 inhibitors (Figure 3). It can conduce to assess the prognosis of patients more intuitively by PNI. We calculated concordance index (C-index), which was 0.777 (95% CI, 0.611-0.944). The calibration curves indicated the reasonable consistency between the predicted and actual survival probability (Figure 4).

## Discussion

Immunotherapy has been approved more indications in patients with advanced NSCLC. From nivolumab monotherapy to pembrolizumab and atezolizumab single or combination therapy, immunotherapy has established its position in the treatment of advanced NSCLC. Several clinical trials have reported durable responses and long-term survival benefits of immunotherapy. However, how to identify the patients most likely to benefit from treatment remains a challenge at present. Therefore, exploring some predictive biomarkers can contribute to selecting the population with better response to immunotherapy, thereby maximizing the benefits of patients.

Although the assessment of tumor and tumor microenvironment is essential to determine biomarkers for immunotherapy, host-related factors, particularly in nutrition and immune status, cannot be ignored. Serum albumin level is a common indicator to evaluate the nutritional status of patients. Low albumin level reflects poor nutritional status, weakens many defense mechanisms of the body, such as cellular and humoral immunity and phagocyte function, and is closely related to the poor prognosis of cancer patients 17, 18. For patients with advanced cancer, pretreatment lymphocytopenia is a poor prognostic factor, which may be associated with pre-existing immunosuppressive conditions. It has been reported that the absolute lymphocyte count level can be used as an alternative indicator to represent the host immune level and predict the overall treatment outcomes in cancer patients 19. PNI integrates serum albumin and lymphocyte levels, which can reflect the host immune-nutritional status of patients. This indicates that it has potential predictive value for advanced NSCLC patients treated with PD-1 inhibitors.

Our study evaluated the early progression and prognosis of advanced NSCLC patients who received PD-1 inhibitors in the clinical practice of our hospital. The results elucidated that pretreatment PNI is a reliable and independent predictor of early progression, PFS, and OS. In the multivariate analysis, the therapy line was also associated with early progression and OS. The predictive role of PNI has been extensively researched in multiple malignancies 20, 21, 22. Preoperative PNI was relevant to postoperative lung complications and long-term prognosis, and can predict the postoperative outcomes of lung cancer in elderly patients 23, 24. Recently, a meta-analysis including 15 articles discussed the prognostic role of PNI in patients with NSCLC 25. The result indicated that low PNI was a reliable indicator of poor OS (*P* < 0.001), and it was also a useful indicator of disease-free survival, recurrence-free survival, and PFS 25. Moreover, previously published studies also suggested that low PNI was a poor prognostic indicator for NSCLC patients who received EGFR-tyrosine kinase inhibitors and was associated with shorter OS 26, 27. Shoji et al. retrospectively analyzed the pretreatment PNI level of 102 NSCLC patients who received ICIs treatment 28. The results suggested that PNI was significantly relevant to the objective response rate (ORR), disease control rate, and PFS in those patients. It also showed a trend as an independent prognostic indicator for OS, but did not reach a statistically significant level (RR, 1.606, *P* = 0.0761) 28. In our study, PNI was a significant independent prognostic indicator for OS (HR, 7.222, 95%CI, 4.081-12.781, *P* < 0.001), and was superior to PFS (HR, 2.698, 95%CI, 1.752-4.153, *P* < 0.001). Overall, PNI is expected to be a simple and novel predictive biomarker for advanced NSCLC patients treated with ICIs, and may help identify patients who will benefit from ICIs treatment.

Our study suggested that the risk of early progression in L-PNI patients was 3.709 times higher than that in H-PNI patients. This may be correlated with the poor nutritional status of patients, the low host immune function, and the inability of PD-1 inhibitors to arouse the anti-tumor effect of the body. Due to the increased risk of early progression in L-PNI patients and the complexity of cancer immunotherapy, the response rate of monotherapy still has considerable space for improvement 5, 6, 29. Therefore, further combination chemotherapy may more benefit in L-PNI patients.

Bone metastasis leads to cancer pain, negative effect on physical conditions and deterioration in quality of life, which also affects the survival outcomes of patients. Recently, Landi et al. evaluated the impact of bone metastases on NSCLC patients treated with nivolumab 30. The results showed that, regardless of the pathological types, ECOG PS status, liver or brain metastases and bone palliative radiotherapy, patients with positive bone metastases had poor ORR, PFS, and OS 30. This suggested that organ-specific metastasis may be a prognostic factor for individual immunotherapy. Our result showed that bone metastasis was a prognostic factor for PFS in the univariate analysis, but did not achieve the same result in multivariate analysis. Besides, ECOG PS status also affected the prognosis of patients treated with immunotherapy 31, 32. Our study concluded that the immunotherapy line was also statistically significant for early progression and survival outcomes of advanced NSCLC patients. It suggested that immunotherapy in initial lines may make advanced NSCLC patients obtain better survival benefits.

Nomogram is a reliable and practical tool for predicting individual survival probability, and its performance is evaluated by consistency index and calibration curves. In this study, we constructed a survival nomogram for 6-, 12- and 18-months OS for advanced NSCLC patients, including the two risk factors of PNI and therapy line. The result suggested that our nomogram of 6- and 12-months had good predictive accuracy.

Despite the advantages of our study, there are also several inevitable limitations that need to be concerned. First of all, this study is a retrospective single-center study with small sample size. Although it can provide certain clinical insights for patient selection, more prospective clinical research are need to verify our conclusions. Besides, insufficient follow-up duration, inconsistent PNI cut-off values, individual differences in nutritional status, comorbidity, and other factors may affect the accuracy of the conclusion. Finally, due to the limited data available, we only evaluated the pretreatment PNI level.

Nevertheless, our research has certain implications for predicting patient clinical outcomes. It can be used as a consideration factor for doctors to choose different patients for ICIs treatment rationally, and can conduce to identify patients with early progression and poor prognosis. The practicability and convenience in the clinic also promote this biomarker to serve clinical decision-making better. Research on the dynamic changes of PNI time-series can also be considered in the future. Besides, exploratory studies related to biomarkers are critical to developing effective approaches to identify and verify the response of ICIs. The prediction model established by PNI combined with different types of biomarkers (such as PD-L1, TMB, and TILs) may be more suitable for clinical application.

## Conclusion

In summary, the present study suggested that PNI is a favorable predictive indicator to evaluate the risk of early progression and survival outcomes in patients with advanced NSCLC during PD-1 inhibitors treatment. Still, prospective studies with further expansion of the sample size are needed to verify and support our conclusions.

## Supplementary Material

Supplementary figures and tables.

## Figures and Tables

**Table 1:** Baseline clinical characteristics of NSCLC patients treated with PD-1 inhibitors

Clinical characteristics	Overall [n (%)]	PNI>46.05[n(%)]	PNI≤46.05 [n (%)]	*P* value
Total	123	70 (56.9)	53 (43.1)	
**Age**				0.898
≤65	82 (66.7)	47 (67.1)	35 (66.0)	
>65	41 (33.3)	23 (32.9)	18 (34.0)	
**Gender**				0.088
Male	98 (79.7)	52 (74.3)	46 (86.8)	
Female	25 (20.3)	18 (25.7)	7 (13.2)	
**Smoking status**				0.946
Never	46 (37.4)	26 (37.1)	20 (37.7)	
Current/previous	77 (62.6)	44 (62.9)	33 (62.3)	
**ECOG PS**				0.101
0	84 (68.3)	52 (74.3)	32 (60.4)	
1-2	39 (31.7)	18 (25.7)	21 (39.6)	
**History**				0.877
LUAD	74 (60.2)	43 (61.4)	31 (58.5)	
LUSC	46 (37.4)	25 (35.7)	21 (39.6)	
Other	3 (2.4)	2 (2.9)	1 (1.9)	
**Stage**				0.611
IIIB/IIIC	23 (18.7)	12 (17.1)	11 (20.8)	
IV	100 (81.3)	58 (82.9)	42 (79.2)	
**Therapy line**				0.604
1	52 (42.3)	31 (44.3)	21 (39.6)	
≥2	71 (57.7)	39 (55.7)	32 (60.4)	
**Immunotherapy drug**				0.753
Nivolumab	22 (17.9)	10 (14.3)	12 (22.6)	
Pembrolizumab	52 (42.3)	30 (42.9)	22 (41.5)	
Sintilimab	31 (25.2)	18 (25.7)	13 (24.5)	
Camrelizumab	10 (8.1)	7 (10.0)	3 (5.7)	
Toripalimab	8 (6.5)	5 (7.1)	3 (5.7)	
**Regimen**				0.055
Monotherapy	27 (22.0)	11 (15.7)	16 (30.2)	
Combination therapy	96 (78.0)	59 (84.3)	37 (69.8)	
**Liver metastasis**				0.254
No	107 (87.0)	63 (90.0)	44 (83.0)	
Yes	16 (13.0)	7 (10.0)	9 (17.0)	
**CNS metastasis**				**0.031**
No	103 (83.7)	63 (90.0)	40 (75.5)	
Yes	20 (16.3)	7 (10.0)	13 (24.5)	
**Bone metastasis**				0.522
No	85 (69.1)	50 (71.4)	35 (66.0)	
Yes	38 (30.9)	20 (28.6)	18 (34.0)	
**PD-L1 expression**				0.962
Positive	24 (19.5)	14 (20.0)	10 (18.9)	
Negative	10 (8.1)	6 (8.6)	4 (7.5)	
Unknown	89 (72.4)	50 (71.4)	39 (73.6)	
**Early progression**				**0.009**
No	99 (80.5)	62 (88.6)	37 (69.8)	
Yes	24 (19.5)	8 (11.4)	16 (30.2)	
**irAEs**				0.694
No	93 (75.6)	52 (74.3)	41 (77.4)	
Yes	30 (24.4)	18 (25.7)	12 (22.6)	

Abbreviation: NSCLC, non-small cell lung cancer; PD-1, programmed death 1; ECOG PS, Eastern Cooperative Oncology Group performance status; LUAD, lung adenocarcinoma; LUSC, lung squamous cell carcinoma; CNS, central nervous system; PD-L1, programmed death-ligand 1; irAEs, immune-related adverse events; PNI, prognostic nutritional index.

**Table 2:** Univariate and multivariate analyses of progression-free survival in NSCLC patients treated with PD-1 inhibitors

Variable	Univariate analysis		Multivariate analysis	
	HR (95%CI)	*P* value	HR (95%CI)	*P* value
Age (≤65 vs. >65)	1.107 (0.715-1.713)	0.649		
Gender (male vs. female)	1.477 (0.911-2.394)	0.113		
Smoking status (never vs. current/previous)	0.842 (0.552-1.283)	0.423		
ECOG PS (0 vs. 1-2)	1.446 (0.940-2.227)	0.094		
History				
LUAD vs. LUSC	1.144 (0.747-1.751)	0.536		
LUAD vs. other	1.001 (0.243-4.124)	0.999		
Stage (IIIB/IIIC vs. IV)	1.117 (0.659-1.896)	0.680		
Therapy line (1 vs. ≥2)	1.918 (1.240-2.966)	**0.003**	1.568 (0.979-2.511)	0.061
Regimen (monotherapy vs. combination therapy)	0.878 (0.535-1.439)	0.605		
Driver genomic alterations (no vs. yes)	1.904 (1.145-3.167)	**0.013**	1.211 (0.683-2.148)	0.512
Liver metastasis (no vs. yes)	1.570 (0.886-2.783)	0.123		
CNS metastasis (no vs. yes)	1.051 (0.603-1.832)	0.859		
Bone metastasis (no vs. yes)	1.577 (1.022-2.434)	**0.040**	1.468 (0.921-2.340)	0.107
PD-L1 expression (no vs. yes)	1.417 (0.514-3.907)	0.500		
irAEs (no vs. yes)	1.089 (0.677-1.752)	0.725		
PNI (high vs. low)	2.798 (1.823-4.292)	**<0.001**	2.698 (1.752-4.153)	**<0.001**

Abbreviation: NSCLC, non-small cell lung cancer; PD-1, programmed death 1; HR, hazard ratio; CI, confidence interval; ECOG PS, Eastern Cooperative Oncology Group performance status; LUAD, lung adenocarcinoma; LUSC, lung squamous cell carcinoma; CNS, central nervous system; PD-L1, programmed death-ligand 1; irAEs, immune-related adverse events; PNI, prognostic nutritional index.

**Table 3:** Univariate and multivariate analyses of overall survival in NSCLC patients treated with PD-1 inhibitors

Variable	Univariate analysis		Multivariate analysis	
	HR (95%CI)	*P* value	HR (95%CI)	*P* value
Age (≤65 vs. >65)	1.306 (0.785-2.175)	0.304		
Gender (male vs. female)	0.910 (0.492-1.684)	0.765		
Smoking status (never vs. current/previous)	1.177 (0.700-1.980)	0.538		
ECOG PS (0 vs. 1-2)	1.856 (1.119-3.080)	**0.017**	1.536 (0.915-2.578)	0.105
History				
LUAD vs. LUSC	1.268 (0.767-2.096)	0.355		
LUAD vs. other	0.584 (0.080-4.265)	0.596		
Stage (IIIB/IIIC vs. IV)	0.974 (0.519-1.828)	0.936		
Therapy line (1 vs. ≥2)	1.898 (1.108-3.254)	**0.020**	2.033 (1.178-3.507)	**0.011**
Regimen (monotherapy vs. combination therapy)	0.624 (0.364-1.071)	0.087		
Driver genomic alterations (no vs. yes)	1.294 (0.689-2.431)	0.423		
Liver metastasis (no vs. yes)	1.292 (0.637-2.620)	0.477		
CNS metastasis (no vs. yes)	1.568 (0.834-2.945)	0.162		
Bone metastasis (no vs. yes)	1.566 (0.941-2.606)	0.085		
PD-L1 expression (no vs. yes)	1.835 (0.514-6.548)	0.350		
irAEs (no vs. yes)	0.734 (0.399-1.352)	0.322		
PNI (high vs. low)	7.596 (4.278-13.486)	**<0.001**	7.222 (4.081-12.781)	**<0.001**

Abbreviation: NSCLC, non-small cell lung cancer; PD-1, programmed death 1; HR, hazard ratio; CI, confidence interval; ECOG PS, Eastern Cooperative Oncology Group performance status; LUAD, lung adenocarcinoma; LUSC, lung squamous cell carcinoma; CNS, central nervous system; PD-L1, programmed death-ligand 1; irAEs, immune-related adverse events; PNI, prognostic nutritional index.

## Abbreviations

PNI: prognostic nutritional index
NSCLC: non-small cell lung cancer
PD-1: programmed death 1
CI: confidence interval
HR: hazard ratio
LC: lung cancer
OS: overall survival
ICIs: immune checkpoint inhibitors
PD-L1: programmed death ligand-1
TMB: tumor mutation burden
TILs: tumor-infiltrating lymphocytes
MMR: mismatch repair
ECOG PS: Eastern Cooperative Oncology Group Performance Status
PFS: progression-free survival
ROC: receiver operating characteristic
K-M: Kaplan-Meier
LUAD: lung adenocarcinoma
LUSC: lung squamous cell carcinoma
EGFR: epidermal growth factor receptor
ROS1: ROS1 proto-oncogene receptor tyrosine kinase
CNS: central nervous system
irAEs: immune-related adverse events
C-index: concordance index
ORR: objective response rate
