# Supplementary material for: Prognostic Nutritional Index identifies risk of early progression and survival outcomes in Advanced Non-small Cell Lung Cancer patients treated with PD-1 inhibitors
Source: J Cancer. 2021 Mar 15;12(10):2960–7. doi: 10.7150/jca.55936 (PMC8040881; doi:10.7150/jca.55936)
Supplement: Supplementary file 1 — Supplementary figures and tables. [file jcav12p2960s1.pdf]

Table S1. Univariate and multivariate logistic analyses of early progression in NSCLC patients treated with PD-1 inhibitors.

Variable	Univariate analysis		Multivariate analysis	
	**OR(95%CI)**	***P* value**	**OR(95%CI)**	***P* value**
Age (≤65 vs. >65)	0.787(0.298-2.083)	0.630		
Gender (male vs. female)	1.039(0.346-3.124)	0.945		
Smoking status (never vs. current/previous)	1.246(0.486-3.191)	0.647		
ECOG PS (0 vs. 1-2)	1.097(0.425-2.833)	0.849		
History				
LUAD vs. LUSC	0.503(0.182-1.387)	0.184		
LUAD vs. other	1.676(0.143-19.639)	0.681		
Stage (IIIB/IIIC vs. IV)	2.962(0.644-13.617)	0.163		
Therapy line (1 vs. ≥2)	6.860(1.922-24.483)	**0.003**	5.860(1.544-22.246)	**0.009**
Regimen (monotherapy vs. combination therapy)	0.808(0.285-2.290)	0.688		
Driver genomic alterations (no vs. yes)	3.308(1.181-9.263)	**0.023**	2.152(0.695-6.667)	0.184
Liver metastasis (no vs. yes)	1.450(0.423-4.968)	0.554		
CNS metastasis (no vs. yes)	1.474(0.477-4.553)	0.500		
Bone metastasis (no vs. yes)	2.256(0.902-5.643)	0.082		
PD-L1 expression (no vs. yes)	1.053(0.168-6.602)	0.956		
irAEs (no vs. yes)	1.361(0.502-3.684)	0.545		
PNI (high vs. low)	3.351(1.307-8.591)	**0.012**	3.709(1.354-10.161)	**0.011**

Abbreviation: NSCLC, non-small cell lung cancer; PD-1, programmed death 1; OR, odds ratio; CI, confidence interval; ECOG PS, Eastern Cooperative Oncology Group performance status; LUAD, lung adenocarcinoma; LUSC, lung squamous cell carcinoma; CNS, central nervous system; PD-L1, programmed death-ligand 1; irAEs, immune-related adverse events; PNI, prognostic nutritional index.

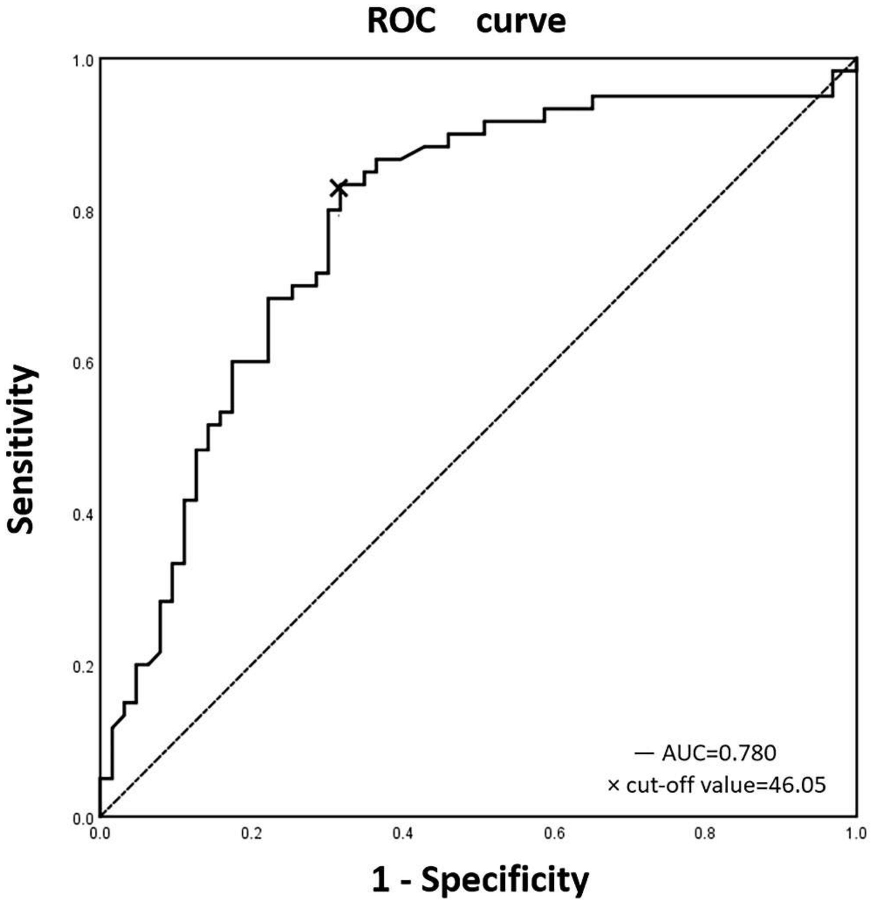

Figure S1. Receiver operating characteristic (ROC) curve analysis of pretreatment prognostic nutritional index (PNI) for overall survival (OS). AUC represents the area under ROC curve (AUC=0.780).

(A)

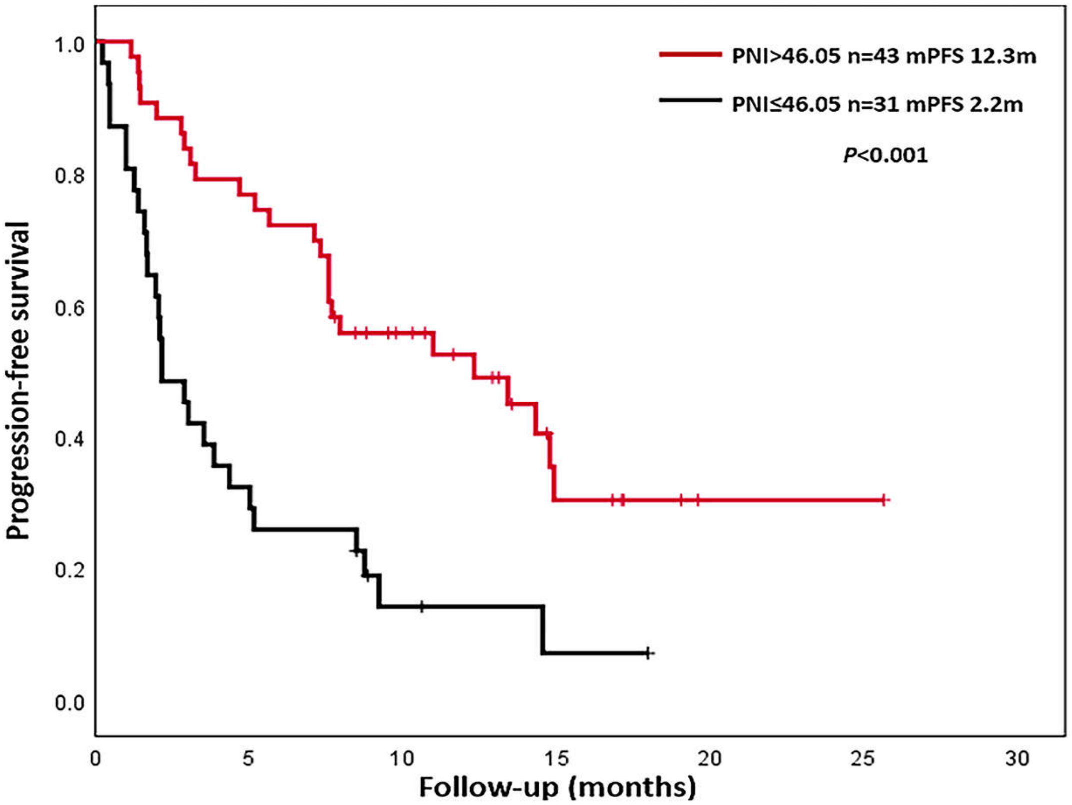
PNI>46.05 n=43 mPFS 12.3m
PNI≤46.05 n=31 mPFS 2.2m
P<0.001
Progression-free survival
1.0
0.8
0.6
0.4
0.2
0.0
0
5
10
15
20
25
30
Follow-up (months)

(B)

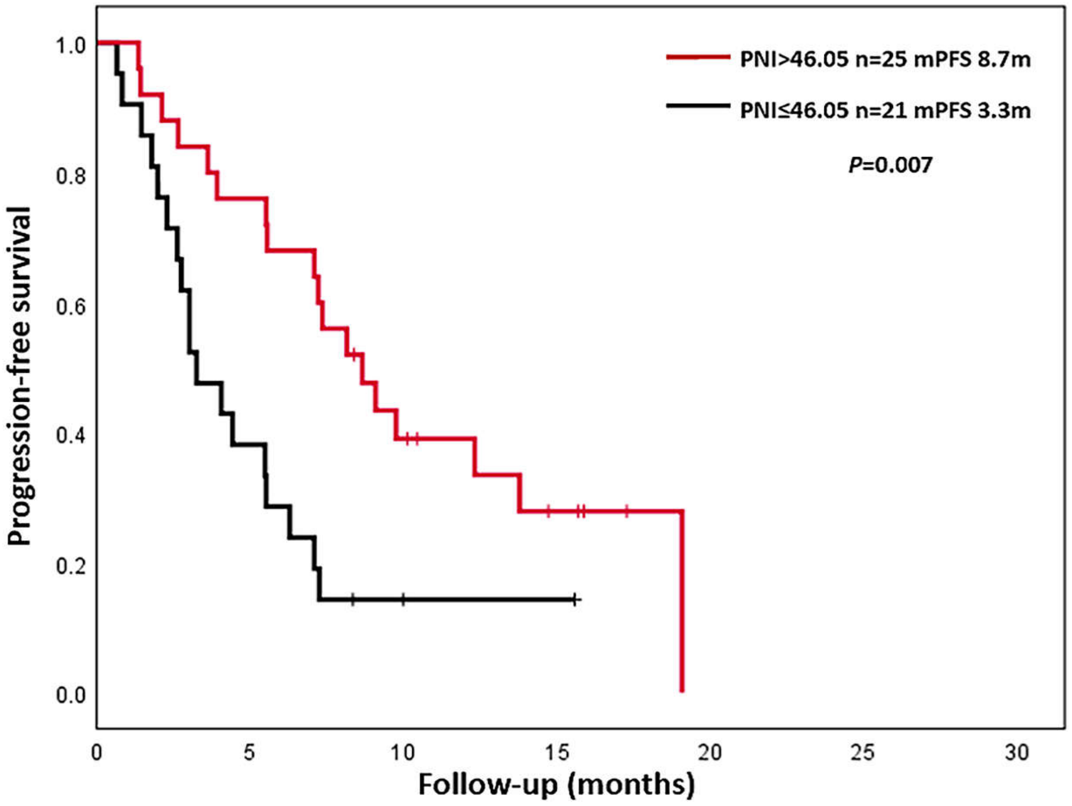
PNI>46.05 n=25 mPFS 8.7m
PNI≤46.05 n=21 mPFS 3.3m
P=0.007
Progression-free survival
1.0
0.8
0.6
0.4
0.2
0.0
0
5
10
15
20
25
30
Follow-up (months)

(C)

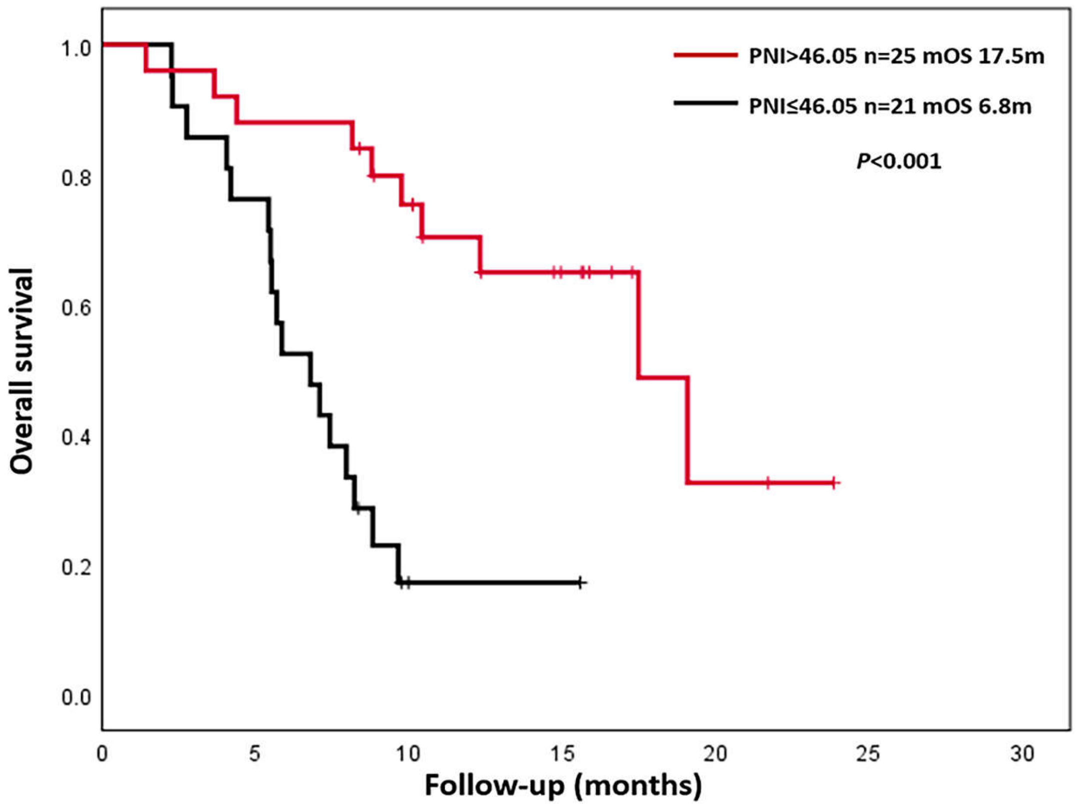

(D)

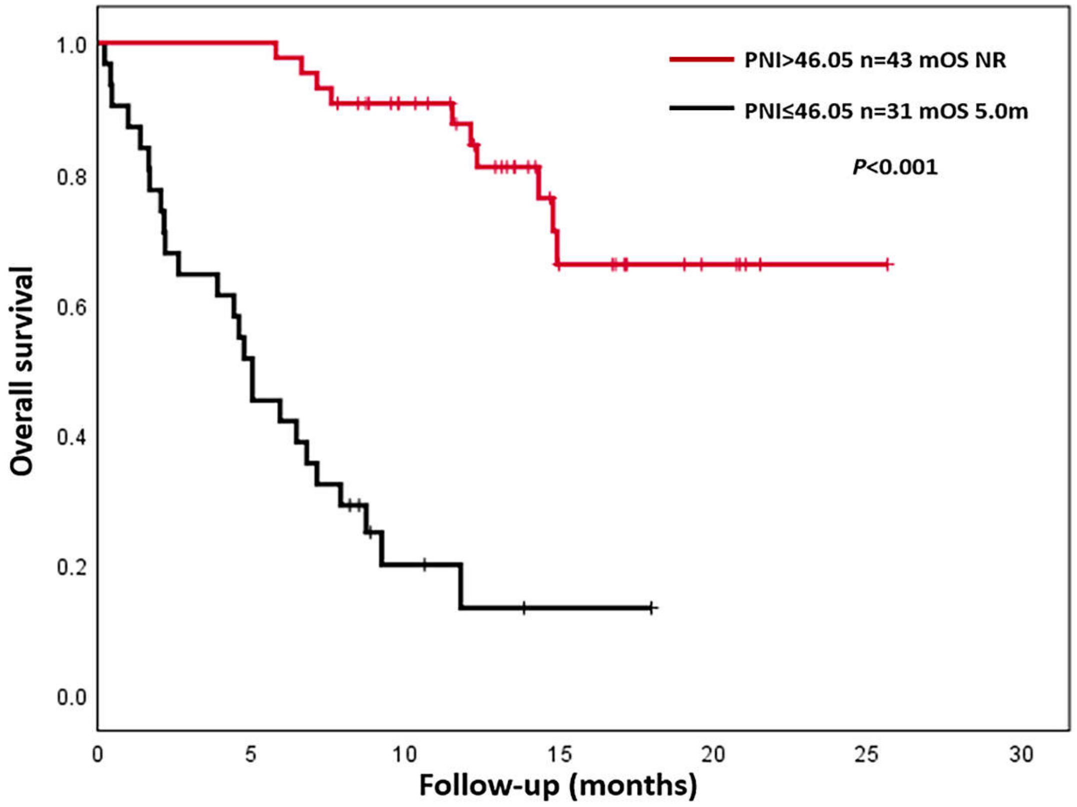

Figure S2. Subgroup analyses of progression-free survival (PFS) and overall survival (OS) with lung adenocarcinoma (LUAD) and lung squamous cell carcinoma (LUSC) patients according to

pretreatment prognostic nutritional index (PNI) level. (A) and (B) described PFS in patients with LUAD and LUSC, respectively. (C) and (D) described OS in patients with LUAD and LUSC, respectively.
